# Clinical outcomes and safety of anakinra in the treatment of multisystem inflammatory syndrome in children: a single center observational study

**DOI:** 10.1186/s12969-023-00858-z

**Published:** 2023-07-31

**Authors:** Brian L.P. Dizon, Christopher Redmond, Emily C. Gotschlich, Sangeeta Sule, Tova Ronis, Kathleen M. Vazzana, Matthew A. Sherman, Rachael Connor, Abigail Bosk, Niti Dham, Ashraf S. Harahsheh, Elizabeth Wells, Roberta DeBiasi, Hemalatha Srinivasalu

**Affiliations:** 1 Division of Rheumatology, Children’s National Hospital, Washington, DC USA; 2 Rheumatology Fellowship and Training Branch, The National Institute of Arthritis and Musculoskeletal and Skin Diseases, Bethesda, MD USA; 3 Department of Pediatrics, George Washington University School of Medicine & Health Sciences, Washington, DC USA; 4 Department of Pediatric Rheumatology, Arnold Palmer Hospital for Children, Orlando, FL USA; 5 Division of Cardiology, Children’s National Hospital, Washington, DC USA; 6 Division of Neurology, Children’s National Hospital, Washington, DC USA; 7 Division of Infectious Diseases, Children’s National Hospital, Washington, DC USA

**Keywords:** Multisystem inflammatory syndrome in children, Cardiac dysfunction, SARS-CoV2, Anakinra, Therapy

## Abstract

**Background and objective:**

Evidence for the treatment of multisystem inflammatory syndrome in children (MIS-C) is lacking. Anakinra, which targets IL-1-mediated inflammation, is reserved for refractory cases of MIS-C; however, its use in the treatment of MIS-C is not clearly established.

**Patients and methods:**

To examine a role for anakinra in MIS-C, we performed a single center observational cohort study of all MIS-C patients diagnosed at our children’s hospital from May 15 to November 15, 2020. Demographics, clinical features, diagnostic testing, and cardiac function parameters were compared between MIS-C patients treated with intravenous immunoglobulin (IVIG) monotherapy and IVIG with anakinra (IVIG + anakinra).

**Results:**

Among 46 patients with confirmed MIS-C, 32 (70%) were in the IVIG + anakinra group, of which 9 (28%) were also given corticosteroids (CS). No patients were treated with anakinra alone. MIS-C patients in the IVIG + anakinra group were enriched in a CV shock phenotype (p = 0.02), and those with CV shock were treated with higher doses of anakinra for a longer duration. Furthermore, MIS-C patients in the IVIG + anakinra group exhibited improvements in fever and cardiac function with or without CS. No significant adverse events were observed, and no differences in IL-1β levels were found among MIS-C patients in the IVIG + anakinra group.

**Conclusions:**

Anakinra treatment, which was co-administered with IVIG primarily in patients with severe MIS-C, was associated with improvements in fever and cardiac function, and demonstrated a favorable side-effect profile. These findings suggest a role for adjunctive anakinra in the treatment of severe MIS-C.

**Supplementary Information:**

The online version contains supplementary material available at 10.1186/s12969-023-00858-z.

## Background

Multisystem inflammatory syndrome in children (MIS-C) is a clinical entity distinct from primary COVID-19 infection that resembles Kawasaki disease (KD) and toxic shock syndrome (TSS) [1–5]. Some features of MIS-C are highly similar to KD, such as persistent fever, hyperinflammation, multiorgan system involvement that commonly includes cardiovascular (CV) dysfunction and coronary artery abnormalities [6–8], and elevated interleukin-1β (IL-1β), as well as IL-6, IL-8, IL-10, and interferon-γ [9, 10]. Given the lack of randomized controlled trials to compare therapeutic approaches used to treat MIS-C, the American College of Rheumatology (ACR) has published consensus treatment guidelines based on expert review, which recommends intravenous immunoglobulins (IVIG) and corticosteroids (CS) as first-line therapy [11]. Biologic medications such as anakinra, which targets IL-1, have been used for refractory cases of MIS-C due to its effectiveness in similar hyperinflammatory diseases such as KD and macrophage activation syndrome (MAS) [12, 13]. IL-1 responses in MIS-C may be elicited by endothelial cell damage from autoantibodies, complement, and immune complexes, lending further support that anakinra may be an effective treatment for MIS-C [2, 11, 14]. Here, we describe the clinical responses and cytokine profiles of a heterogeneous cohort of confirmed MIS-C patients treated with IVIG monotherapy and IVIG with anakinra. Our findings reveal favorable clinical outcomes associated with treatment of MIS-C patients with adjunctive anakinra, suggesting that anakinra may be a safe and efficacious treatment for severe MIS-C.

## Methods

### Study design, setting, and subjects

This observational clinical cohort study included children as they were hospitalized for MIS-C at our children’s hospital over a 6-month period (May 15 to November 15, 2020). The study received approval by the institutional research board, and a waiver of informed consent was granted. The clinical descriptions, diagnostic testing, and therapies for confirmed MIS-C patients were extracted from the electronic health record as previously described [14].

### Treatment algorithm

A multidisciplinary team at our children’s hospital was created to respond quickly to suspected cases of MIS-C through daily meetings and the creation of protocol for the evaluation and treatment of MIS-C as previously described [14]. The protocol was developed and implemented prior to the publication of ACR guidelines for management of MIS-C. All patients who met the CDC case definition for MIS-C were hospitalized and treated with IVIG at 2 g/kg and aspirin as recommended by the ACR guidelines for the management of MIS-C [11]. Using the institutional algorithm, anakinra was initiated at the clinical judgement of the pediatric rheumatologist in conjunction with the hospitalist team and consultants in the following situations: (1) as adjunct immunomodulatory intervention for MIS-C in critically-ill patients admitted to ICU, or (2) as first-line rescue therapy for those refractory to IVIG. Anakinra was chosen for treatment of MIS-C by virtue of its IL-1 targeted mechanism of action, short half-life, and titratable effects [12, 15]. An algorithm for starting doses of anakinra and its up-titration in MIS-C patients was previously reported [14]. Briefly, intravenous anakinra was started at 8-10 mg/kg/day divided every 6 h in critically ill patients and 6-8 mg/kg/day divided every 6 h in non-critically ill patients at the clinical judgement of the consulting pediatric rheumatologist [14]. Weaning of intravenous anakinra was accomplished by reducing the dose by ~ 2 mg/kg/day every 24–48 h in clinically stable patients until discontinued before hospital discharge (Supplemental Figure S1).

### Outcome measurements

Echocardiograms (echo) were performed with either Phillips (Andover, MA) or GE (Chicago, IL) vendor machines as previously reported [6, 16]. Conventional echocardiographic measurements were made according to American Society of Echocardiography guidelines, including left ventricular ejection fraction (LVEF) by modified Simpson’s biplane method. Normal LVEF was defined as ≥ 55%. Cardiac longitudinal strain (apical four-chamber view, or Ap4) was measured off-line on an independent vendor platform, TomTec. A normal value of -21.4 was extrapolated from adult data and accepted by the American Society of Echocardiography [17]. Recorded temperature from routine vital sign measurements during hospitalization was extracted from the patient charts, and fever defervescence was defined as the first measurement of oral or rectal temperature < 38.0 °C. Adverse events such as neutropenia, rash, elevated liver function tests, hemolytic anemia, anaphylaxis, and re-hospitalization, were extracted from the patient charts and defined according to Common Terminology Criteria for Adverse Events (CTCAE) version 5.0.

### Cytokine profiles in MIS-C

To explore a physiologic basis for the clinical outcomes in MIS-C patients who received adjunctive anakinra, cytokine profiles in blood samples collected shortly after admission but prior to treatment with anakinra were analyzed. Due to limited data from patients who received IVIG + anakinra with CS, analysis of cytokine profiles according to clinical phenotype was made between patients selected to receive IVIG monotherapy or IVIG + anakinra treatment.

### Statistical analyses

Continuous data were summarized using descriptive statistics of medians with 25th and 75th percentiles. Categorical variables were described as percentages and analyzed by Fisher’s exact test. Normal ranges for laboratory tests performed at our institution were compiled in Supplemental Table S1. No imputations were made for missing data (Supplemental Figure S2). Non-parametric Mann Whitney test was used when comparing continuous measures between patients with/without KD-like features, CV shock, and anakinra treatment. Paired longitudinal echocardiographic measurements were analyzed with a Wilcoxon matched pairs signed rank test. Statistical analysis of defervescence after treatment initiation for MIS-C was calculated with log rank test. For all analyses, a non-adjusted p-value < 0.05 was considered significant given the exploratory nature of the study. The data analysis plan was discussed with biostatisticians at our children’s hospital. All analyses were performed with Prism 9 software, and Adobe Illustrator 2022 was used for graphical representations of the data.

## Results

### Patient demographics

During the study period, 46 patients who satisfied the 2020 CDC MIS-C surveillance case definition and whose final diagnoses were confirmed by our MIS-C Task Force were identified and further categorized by the presence/absence of KD-like features, as defined by the American Heart Association [13], and CV shock, which was defined as persistent fluid-refractory hypotension requiring vasoactive support as previously reported [14]. MIS-C patients without KD-like features and CV shock were termed non-specific [18], while those with both KD-like features and CV shock were termed Kawasaki disease shock syndrome-like (KDSS-like).

Of the 46 patients in the study, 14 (30%) received IVIG monotherapy. The remaining 32 (70%) patients received IVIG and anakinra (referred to as IVIG + anakinra), and these patients were further subdivided into those given CS (with CS), which included stress hydrocortisone (19%) and inflammatory steroid (9%), and those who were not given CS (without CS) (72%) (Table 1). The median age at diagnosis was 8 [4 − 13] years, and 54% were male (Table 1). CV shock was present in 65% of MIS-C patients in the IVIG + anakinra group (vs. 21% in the IVIG monotherapy group, p = 0.02) (Table 1). 34% of MIS-C patients in the IVIG + anakinra group presented with KDSS (vs. 7% in the IVIG monotherapy group, p = 0.07) (Table 1).

**Table 1:** Comparison of MIS-C patients in the IVIG monotherapy versus IVIG + anakinra groups

Parameter	All MIS-C	IVIG monotherapy	All IVIG + anakinra	p-value
Patients (n, %)	46, 100%	14, 30%	32, 70%	N/A
**Demographics**				
Age, in years [Q1, Q3]	8 [4,13]	10.5 [3.5, 13]	8 [4, 10]	0.57
Male (n, %)	25, 54%	8, 57%	17, 53%	> 0.99
African American (n, %)	25, 54%	8, 57%	17, 53%	> 0.99
Latin-American (n, %)	20, 43%	6, 43%	14, 44%	> 0.99
Caucasian (n, %)	1, 2%	0, 0%	1, 3%	> 0.99
**MIS-C clinical features**				
SARS-CoV2 PCR + (n, %)	19, 41%	5, 36%	14, 44%	0.75
Non-specific (n, %)	15, 33%	9, 64%	6, 19%	0.005
KD-like (n, %)	20, 43%	3, 21%	17, 53%	0.06
CV shock (n, %)	23, 50%	3, 21%	20, 63%	0.02
KDSS-like (n, %)	12, 26%	1, 7%	11, 34%	0.07
**Medications**				
IVIG (n, %)	46, 100%	14, 100%	32, 100%	N/A
Inflammatory steroids (n, %)	3, 7%	0, 0%	3, 9%	0.54
Stress hydrocortisone (n, %)	7, 15%	1, 7%	6, 19%	0.41

### Characteristics of MIS-C patients who received IVIG+anakinra

The median dose of anakinra in the IVIG + anakinra group was 7.9 [7.5–8.2] mg/kg/day, with a median maximum dose of 9.5 [7.7–10.0] mg/kg/day, and a median duration of 9.8 [7.0–11.3] days (Supplemental Table S3). 100% (20/20) of patients with CV shock were given anakinra as adjunctive immunosuppression, and 100% (12/12) of patients without CV shock received anakinra as rescue therapy (Table 2). Among all MIS-C patients in the IVIG + anakinra group, those with CV shock presented with high median troponin (0.05 vs. 0.02 ng/mL without CV shock; normal < 0.04 ng/mL, p = 0.03) and BNP (3339 vs. 1472 pg/mL without CV shock; normal < 1157 pg/mL, p = 0.04) (Supplemental Table S4). Patients with CV shock received doses of anakinra at 9.8 mg/kg/day (vs. 8.0 mg/kg/day without CV shock, p = 0.003) for a treatment duration of 10.6 days (vs. 7.1 days without CV shock, p = 0.002) (Table 2).

**Table 2:** Comparison of MIS-C patients in the IVIG + anakinra group presenting with or without CV shock

Parameter	- CV shock	+ CV shock	p-value
Patients (n, %)	12, 38%	20, 63%	N/A
**Medications**			
Inflammatory steroids (n, %)	0, 0%	3, 15%	0.27
Stress hydrocortisone (n, %)	0, 0%	6, 30%	0.06
**Anakinra treatment characteristics**			
Adjunct therapy (n, %)	0, 0%	20, 100%	N/A
First-line rescue therapy (n, %)	12, 100%	0, 0%	N/A
Starting dose, mg/kg/day [Q1, Q3]	7.7 [4.5, 8.0]	7.9 [7.7, 9.9]	0.09
Initiation, hospitalization day [Q1, Q3]	2.0 [2.0, 4.8]	2.0 [1.0, 3.0]	0.11
Max dose, mg/kg/day [Q1, Q3]	8.0 [4.5, 9.2]	9.8 [8.1, 10.0]	0.003
Max dose, mg/dose [Q1, Q3]	41 [30, 94]	55 [46, 100]	0.21
Treatment duration, days [Q1, Q3]	7.1 [4.6, 8.8]	10.6 [9.7, 13.3]	0.002

### Resolution of fever in MIS-C patients

**Fig. 1:**
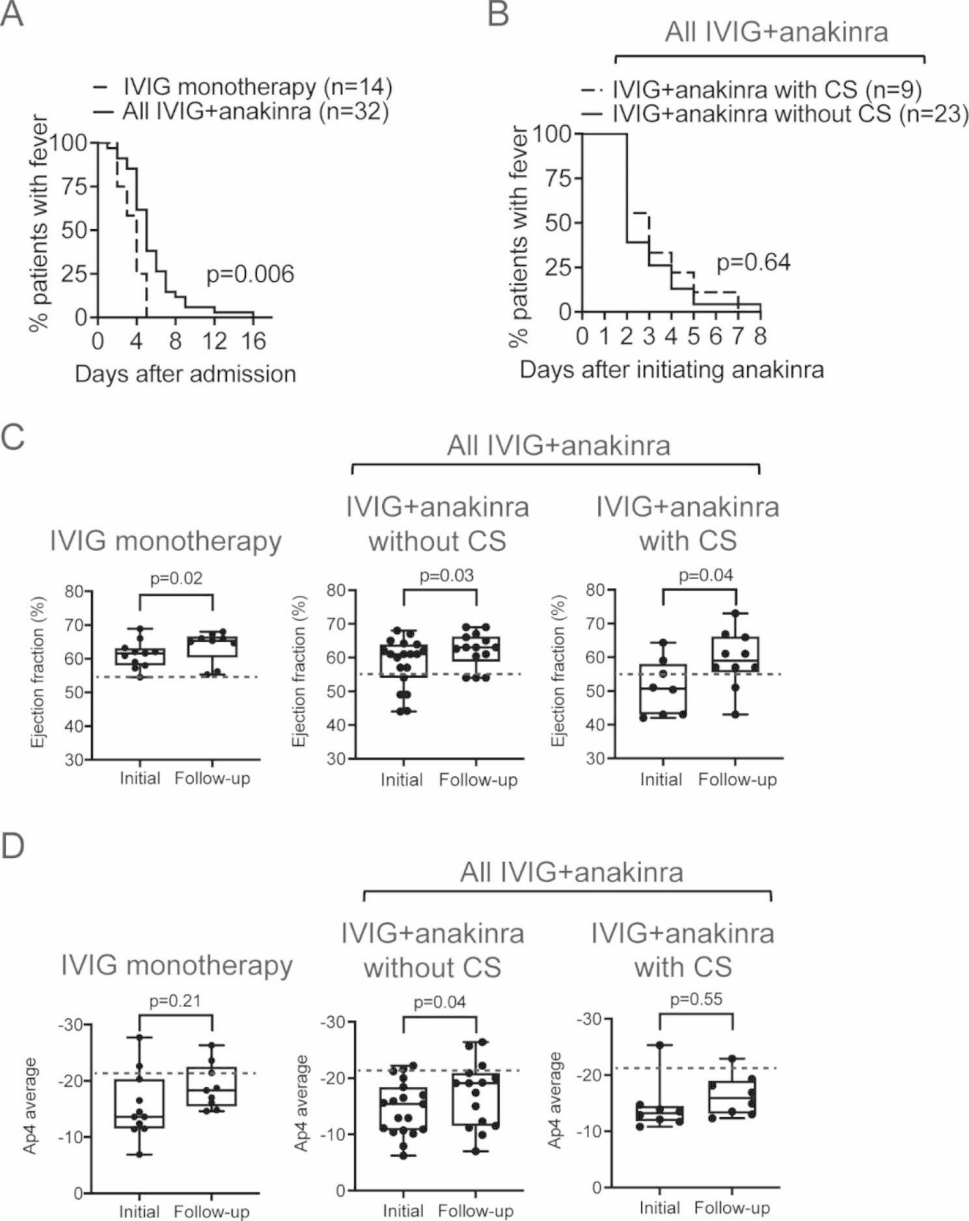
Clinical outcomes associated with treatment of MIS-C patients Effects of anakinra on fever and cardiac function in MIS-C patients. Fever in patients given IVIG monotherapy or IVIG + anakinra starting from admission **(A)** and in IVIG + anakinra patients treated with or without CS **(B)** are shown. LVEF **(C)** and cardiac strain **(D)** are shown among the treatment groups. Dotted lines denote the cut-off ranges for normal LVEF (55%) and cardiac strain (-21.4). Data were analyzed by log rank test (A and B) or non-parametric two-tailed Wilcoxon matched pairs signed rank test (C and D), and a p-value < 0.05 was considered statistically significant

Resolution of fever occurred in 100% of MIS-C patients receiving IVIG monotherapy by 5 days after admission, compared to patients in the IVIG + anakinra group, with ~ 50% defervescence occurring by 5 days after admission (p = 0.003) (Fig. 1A and B). Stress or inflammatory dose CS were used in 9 patients (28%) in the IVIG + anakinra group, and no significant differences in fever resolution were observed between MIS-C patients with CS and without CS (p = 0.64) (Fig. 1B). Subgroup analysis revealed that among patients with KD-like or KDSS-like features in the IVIG + anakinra group, complete defervescence occurred by 7 days after anakinra treatment was initiated (Supplemental Fig. 2 C and 2E).

### Longitudinal cardiac function measurements in MIS-C patients

The median time between initial and follow-up echo after treatment initiation was 11 [9–14] days (Supplemental Figure S3B). No patients in the IVIG monotherapy group exhibited a reduced LVEF < 55% in their initial echo, although a statistically significant increase in LVEF was measured in the follow-up compared to the initial echo (62% vs. 65%, p = 0.01) (Fig. 1C). 10 patients (37%) in the IVIG + anakinra group exhibited reduced LVEF (< 55%) at baseline. LVEF in these patients increased in the follow-up echo after treatment (59.0% vs. 62.5%, p = 0.004) (Fig. 1C). MIS-C patients who received CS in addition to IVIG + anakinra exhibited an increase in LVEF from initial to follow-up (51 to 57%, p = 0.04) (Fig. 1C). Analysis of cardiac strain by Ap4 in MIS-C patients in the IVIG + anakinra group improved in follow-up compared to the initial echo (-13.2 to -17.8, p = 0.04). No improvements were seen in the IVIG monotherapy (-15.5 to -18.9, p = 0.21) or IVIG + anakinra with CS groups (-14.6 to -16.1, p = 0.55) (Fig. 1D). MIS-C patients in the IVIG + anakinra group with CV shock and KDSS-like features exhibited improvements in LVEF on follow-up compared to initial echo (49 to 59% for CV shock, p = 0.005; 49 to 59% for KDSS-like, p = 0.047) (Supplemental Figure S2G). MIS-C patients in the IVIG + anakinra group with KD-like features also had improved Ap4 cardiac strain on follow-up compared to initial echo (-14.4 to -18.4, p = 0.005) (Supplemental Figure S2I).

### Adverse events

No mortality or thrombosis events were observed in the entire cohort of MIS-C patients (0/46 or 0%). Patients in the IVIG + anakinra group had increased percentages of neutropenia (20% vs. 7% in IVIG monotherapy, p = 0.25) and elevated liver function tests (LFTs) (24% vs. 14% in IVIG monotherapy, p = 0.25) (Table 3). No significant differences in hemolytic anemia, anaphylaxis, or re-hospitalization were found between MIS-C patients in the IVIG monotherapy and IVIG + anakinra without CS groups (Table 3). Patients who received IVIG + anakinra with CS had a higher incidence of elevated LFTs (Table 3), but the degree of elevation was mild in most of the patients (CTCAE grade < 2) (Supplemental Table S5).

**Table 3:** Adverse events associated with IVIG + anakinra treatment in MIS-C patients

Adverse event	IVIG monotherapy (n = 14)	IVIG + anakinra without CS (n = 25)	IVIG + anakinra with CS (n = 9)	p-value
Elevated LFTs (n %)	2, 14%	6, 24%	7, 67%	0.008*
Neutropenia (n %)	1, 7%	5, 20%	3, 33%	0.36
Hemolytic anemia (n %)	1, 7%	1, 4%	1, 11%	0.73
Anaphylaxis (n %)	0, 0%	0, 0%	0, 0%	N/A
Rash (n %)	0, 0%	2, 8%	0, 0%	0.42
Re-hospitalization (n %)	0, 0%	0, 0%	0, 0%	N/A

### Comparison of cytokine profiles in MIS-C patients in the IVIG monotherapy and IVIG + anakinra groups

Due to limited data from patients who received IVIG + anakinra with CS, analysis of cytokine profiles was limited to patients selected to receive IVIG monotherapy or IVIG + anakinra. No significant differences in circulating levels of IL-1β in the cohort of MIS-C patients were found (p = 0.37) (Table 4). Patients in the IVIG + anakinra group had elevated circulating levels of pro-inflammatory markers including sIL-2R (5.7 vs. 1.4 fold difference, p = 0.0006), IL-6 (22.1 vs. 3.0 fold difference in IVIG monotherapy, p = 0.006), and IL-8 (1.0 vs. 0.3 fold difference in IVIG monotherapy, p = 0.03) (Table 4).

**Table 4:** Comparison of circulating cytokines in MIS-C patients

Cytokines, fold difference [Q1, Q3]	All MIS-C	IVIG monotherapy	All IVIG + anakinra	p-value
IFNγ	1.0 [0.4–1.6]	0.4 [0.40–1.2]	1.0 [0.5–2.1]	0.07
IL-1β	0.9 [0.2–1.0]	0.9 [0.7–0.9]	0.9 [0.2–1.0]	0.37
IL-2	1.0 [1.0–1.0]	1.0 [1.0–1.0]	1.0 [1.0–1.0]	0.74
IL-6	14.3 [3.2–34.4]	3.0 [1.6–15.8]	22.1 [9.9–39.0]	0.006
IL-8	0.6 [0.3–1.0]	0.3 [0.3–0.6]	1.0 [0.3–1.0]	0.03
IL-12	0.4 [0.4–1.0]	0.4 [0.4–0.4]	0.7 [0.4–1.0]	0.10
IL-17	1.0 [0.6–1.6]	1.0 [0.6–3.0]	1.0 [0.6–1.0]	0.98
sIL-2R	4.0 [2.0–6.7]	1.4 [0.9–3.2]	5.7 [3.7–7.8]	0.0006
TNFα	0.3 [0.1–0.5]	0.2 [0.1–0.3]	0.3 [0.2–0.6]	0.11

## Discussion

### The short-term outcomes of MIS-C patients treated with IVIG + anakinra were favorable

The American College of Rheumatology (ACR) MIS-C Task Force treatment recommendations vary based on disease severity, ranging from no treatment for mild disease, IVIG and steroid for moderate and severe disease, and anakinra for signs of MAS [11]. In keeping with these recommendations, anakinra was used as adjunctive therapy in place of CS in patients with severe or refractory MIS-C in this study. This allowed for a clearer observation of anakinra’s effect on MIS-C without the confounding effects of CS.

In this cohort, MIS-C patients treated with IVIG + anakinra had improved fever and cardiac function (Fig. 1). In the relatively short follow-up period, improvements in LVEF were seen in MIS-C patients treated with IVIG + anakinra, both with and without CS, supporting the rapid and significant impact of anakinra on severe MIS-C. The smaller improvement in cardiac strain seen in MIS-C is consistent with prior observations that LVEF normalizes more quickly than other measures of cardiac function, such as cardiac strain [16]. These findings are similar to treatment responses reported for patients with MIS-C who received CS monotherapy or IVIG with CS [19–25]. Numerous studies have reported effective defervescence and normalization of LVEF in MIS-C patients who received anakinra [26–28]; however, the contribution of anakinra in these reports is difficult to assess due to concurrent CS use in most of the patients. Given the observational nature of this cohort study, variability in disease severity between the treatment groups, and the lack of a comparator group that received CS without anakinra, we are unable to make a direct comparison of disease outcomes between anakinra and CS. However, MIS-C patients who received CS in addition to IVIG + anakinra exhibited similar clinical responses as measured by fever resolution and cardiac function when compared to those who received IVIG + anakinra only without CS (Fig. 1B and G). Overall, these findings suggest that anakinra is an effective adjunctive treatment for patients with severe manifestations of MIS-C, such as CV shock. However, more rigorous evaluation through clinical trials are needed to determine whether IVIG + anakinra is superior to IVIG with CS.

Analysis of adverse reactions between patients in the IVIG monotherapy and IVIG + anakinra groups revealed a number of key observations. MIS-C patients treated according to our hospital’s treatment algorithm had no thromboses or deaths, which have been reported in earlier MIS-C cohorts receiving IVIG with or without CS (Table 3) [19, 20, 23]. The patients who received CS with IVIG + anakinra exhibited only mild elevations in LFTs when compared to those who received IVIG monotherapy (Supplemental Table 5). MIS-C patients in our cohort were hospitalized for a median of 11 [9–14] days (Table 2) and were discharged only after weaning off all immunomodulatory agents. It is difficult to compare hospital length-of-stay between patients receiving IVIG monotherapy and IVIG + anakinra since MIS-C patients with more severe disease were selected to receive anakinra, which likely contributed to longer hospitalization. However, it is notable that no patients treated with anakinra were re-admitted for recurrence of MIS-C symptoms after discharge, as previously reported in patients treated with IVIG and CS [29]. Taken together, these findings suggest that anakinra is a potentially safe and effective treatment for MIS-C, although further studies are needed to compare the overall clinical outcomes and cost effectiveness of anakinra treatment compared to CS and other biologics. Lastly, while the cases of MIS-C worldwide have declined [30], possibly from vaccination against SARS-CoV-2 and differences in clinical severity by COVID-19 variants [31, 32], the results of this study regarding the safety and efficacy of intravenous anakinra in the context of MIS-C can potentially be extended to other hyperinflammatory rheumatologic conditions, such as systemic JIA and MAS.

### The use of anakinra for treatment of MIS-C suggests a role for IL-1-mediated inflammatory pathways

Defervescence associated with anakinra treatment suggested that IL-1 plays a role in the pathophysiology of MIS-C. Interestingly, in contrast to IL-6 which was significantly elevated in this cohort, no elevation in serum IL-1β levels was seen (Table 4). The lack of elevated serum IL-1β in patients with MIS-C is consistent with other reports [33, 34]. While serum IL-1β is repeatedly normal in MIS-C, IL-1β gene expression has been previously shown to be higher in MIS-C compared to KD [35], suggesting IL-1β may exist in a bound state on the surface of immune cells or act locally within tissue to modulate inflammatory responses in MIS-C [36].

### Limitations of study

The observational clinical design of this study prevented direct comparison of different treatments. The use of an institutional algorithm introduced a selection bias for patients with more severe features of MIS-C to receive anakinra, and the decision to initiate anakinra in MIS-C patients was based on the clinical impression of the in-patient team and not on objective lab tests. Analysis of data from a small cohort of patients from a single academic institution was counterbalanced by the granularity of data in a clearly defined and systematically treated population of patients with MIS-C. Our study may have been underpowered to detect differences in side-effect profiles of anakinra. Lastly, this study included patients and data limited to MIS-C diagnosed during the first wave of SARS-CoV2 in Washington DC, during which Alpha was the predominant variant, and may not represent MIS-C caused by other SARS-CoV2 variants, such as Delta and Omicron.

## Conclusions

The addition of anakinra to IVIG for treatment of the multiple clinical phenotypes of MIS-C was associated with favorable outcomes, including fever resolution and cardiac function with minimal adverse effects.

## Electronic supplementary material

Below is the link to the electronic supplementary material.

Supplementary Material 1

Supplementary Material 2

## Data Availability

Deidentified individual participant data are not publicly available due to ongoing collection and analysis but are available from the corresponding author on reasonable request.

## Abbreviations

MIS-C: Multisystem inflammatory syndrome in children
IVIG: Intravenous immunoglobulin
LVEF: Left ventricular ejection fraction
KD: Kawasaki disease
CV: Cardiovascular
TSS: Toxic shock syndrome
KDSS: Kawasaki disease shock syndrome
MAS: Macrophage activation syndrome
ACR: American College of Rheumatology
Ap4: Apical four-chamber view
ALC: Absolute lymphocyte count
CS: Corticosteroids
