# Supplementary material for: Clinical outcomes and safety of anakinra in the treatment of multisystem inflammatory syndrome in children: a single center observational study
Source: Pediatr Rheumatol Online J. 2023 Jul 31;21:76. doi: 10.1186/s12969-023-00858-z (PMC10388456; doi:10.1186/s12969-023-00858-z)
Supplement: Supplementary file 1 — Supplementary Material 1 [file 12969_2023_858_MOESM1_ESM.docx]

**SUPPLEMENT CONTENTS**

Supplemental Figure S1. Algorithm for weaning anakinra in MIS-C patients.

Supplemental Table S1. Normal ranges for laboratory tests in MIS-C patients included in the study.

Supplemental Table S2. Available cytokine results from MIS-C patients included in the study.

Supplemental Table S3. Comparison of MIS-C patients in the IVIG monotherapy and IVIG+anakinra groups (continued).

Supplemental Table S4. Comparison of MIS-C patients in the IVIG+anakinra group presenting with or without CV shock (continued).

Supplemental Figure S2. Clinical outcomes of MIS-C patients.

Supplemental Figure S3. Timing of initial and follow-up echocardiograms in children diagnosed with MIS-C.

Supplemental Table S5. Adverse events of severity of neutropenia and elevated LFTs stratified by severity according to CTCAE.


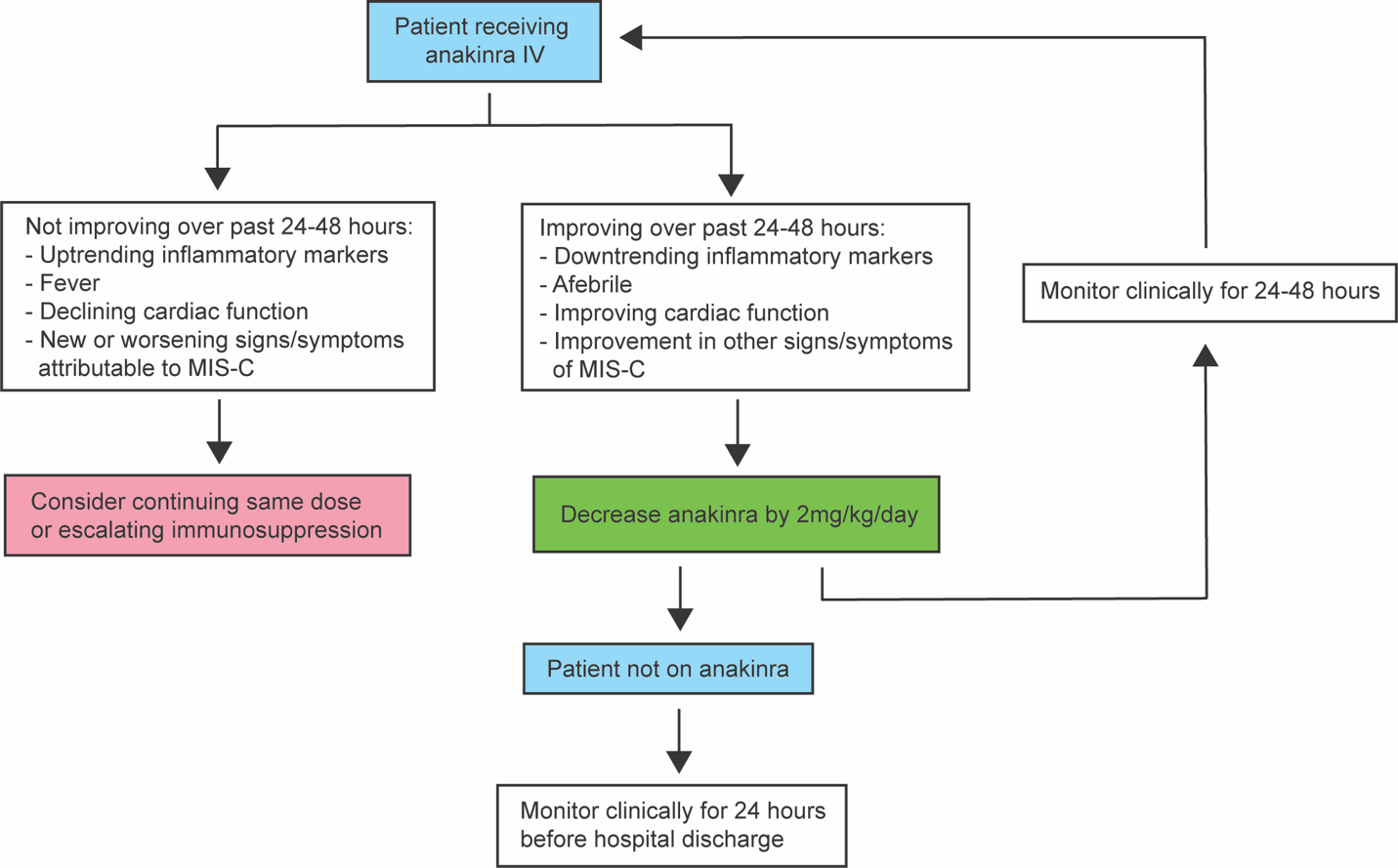


**Supplemental Figure S1. Algorithm for weaning anakinra in MIS-C patients.** The weaning algorithm for MIS-C patients treated with anakinra is shown. In MIS-C patients treated with anakinra who demonstrated objective evidence of clinical improvement, anakinra was decreased by 2mg/kg/day in 24-48 hour increments with continued clinical monitoring until its discontinuation.

| **Laboratory test** | **Units** | **Normal range** | **Cut-off for abnormal** |
| --- | --- | --- | --- |
| Albumin | g/dL | 3.5 – 5.6 | < 3.5 |
| ALT | units/L | 24 – 59 | > 59 |
| AST | units/L | 16 – 57 | > 57 |
| BNP | pg/mL | < 1157 | > 1158 |
| Cr | mg/dL | 0.2 – 0.79 | > 0.79 |
| CRP | mg/dL | 0.05 – 1.00 | > 1.00 |
| D-dimer | mg/mL | <0.57 | > 0.57 |
| Ferritin | ng/mL | 10 – 500 | > 500 |
| Fibrinogen | mg/dL | 179 – 469 | > 469 |
| Hgb | g/dL | 10.2 – 13.4 | < 9 |
| INR | N/A | 0.91 – 1.09 | > 1.09 |
| LDH | units/L | 155-280 | > 280 |
| Lymphocytes (ALC) | K/mcL | 1.25 – 5.77 | Lymphopenia <1.2 |
| Neutrophils (ANC) | K/mcL | 1.54 – 8.29 | Neutropenia < 1.5  Neutrophilia > 7.7 |
| Platelets | K/mcL | 189 - 459 | Thrombocytopenia < 150  Neutrophilia > 450 |
| Troponin | ng/mL | < 0.04 | 0.04 |
| WBC | K/mcL | 3.84 – 13.38 | Leukopenia < 3.5  Leukocytosis > 15 |

**Supplemental Table S1. Normal ranges for laboratory tests in MIS-C patients included in the study.** All laboratory tests analyzed for the study are shown with units, and the defined cut-offs for abnormal values are indicated.

| **Parameter** | **All MIS-C** | **All KD-like** | **All CV shock** |
| --- | --- | --- | --- |
| Patients (n, %) | 46, 100% | 20, 44% | 22, 48% |
| TNFα (n, %) | 34, 76% | 15, 83% | 18, 82% |
| IL-2 (n, %) | 34, 76% | 15, 83% | 18, 82% |
| sIL-2R (n, %) | 25, 56% | 8, 44% | 13, 59% |
| IL-12 (n, %) | 26, 58% | 8, 44% | 13, 59% |
| IFNγ (n, %) | 34, 76% | 15, 83% | 18, 82% |
| IL-4 (n, %) | 35, 78% | 15, 83% | 18, 82% |
| IL-5 (n, %) | 35, 78% | 15, 83% | 18, 82% |
| IL-10 (n, %) | 34, 76% | 15, 83% | 18, 82% |
| IL-13 (n, %) | 25, 56% | 8, 44% | 13, 59% |
| IL-17 (n, %) | 25, 56% | 8, 44% | 13, 59% |
| IL-1β (n, %) | 35, 78% | 15, 83% | 18, 82% |
| IL-6 (n, %) | 37, 82% | 17, 94% | 19, 86% |
| IL-8 (n, %) | 33, 73% | 14, 78% | 18, 82% |

**Supplemental Table S2. Available cytokine results from MIS-C patients included in the study.** The available cytokine results for MIS-C patients are indicated as the absolute number and percentage.

| **Parameter** | **All MIS-C patients** | **IVIG monotherapy** | **All IVIG+anakinra** | **p-value** |
| --- | --- | --- | --- | --- |
| Patients (n, %) | 46, 100% | 14, 30% | 32, 70% | N/A |
| **Anakinra treatment characteristics** |  |  |  |  |
| Starting dose, mg/kg/day [Q1, Q3] | N/A | N/A | 7.9 [7.5 – 8.2] | N/A |
| Initiation, hospitalization day, [Q1, Q3] | N/A | N/A | 2.0 [1.3 – 3.0] | N/A |
| Max dose, mg/kg/day [Q1, Q3] | N/A | N/A | 9.5 [7.7 – 10.0] | N/A |
| Max dose, mg/dose [Q1, Q3] | N/A | N/A | 51 [40 – 99] | N/A |
| Treatment duration, days [Q1, Q3] | N/A | N/A | 9.8 [7.0 – 11.3] | N/A |
| **Laboratory findings on admission** |  |  |  |  |
| D-dimer, mg/mL [Q1, Q3] | 3.1 [1.7 – 4.8] | 2.9 [1.2 – 3.2] | 3.1 [1.7 – 5.5] | 0.41 |
| Albumin, g/dL [Q1, Q3] | 3.0 [2.6 – 3.5] | 3.0 [2.6 – 3.6] | 2.0 [2.6 – 3.5] | 0.69 |
| Fibrinogen, mg/dL [Q1, Q3] | 518 [409 – 617] | 495 [387 – 598] | 520 [426 – 620] | 0.44 |
| LDH, units/L [Q1, Q3] | 286 [232 – 345] | 246 [185 – 409] | 292 [256 – 345] | 0.30 |
| INR, units [Q1, Q3] | 1.2 [1.1 – 1.3] | 1.3 [1.1 – 1.4] | 1.2 [1.1 - 1.3] | 0.91 |
| ALT, units/L [Q1, Q3] | 37 [18 – 70] | 23 [14 – 62] | 42 [20 – 71] | 0.28 |
| AST, units/L [Q1, Q3] | 39 [25 – 59] | 32 [23 – 55] | 43 [27 – 60] | 0.29 |
| Cr, mg/dL [Q1, Q3] | 0.49 [0.39 – 0.66] | 0.49 [0.30 – 0.68] | 0.49 [0.40 – 0.68] | 0.56 |
| **Cytokine measurements** |  |  |  |  |
| IL-4 (fold Δ [Q1, Q3]) | 0.88 [0.88 – 1.0] | 0.88 [0.88 – 0.94] | 0.94 [0.88 – 1.0] | 0.23 |
| IL-5 (fold Δ [Q1, Q3]) | 1.0 [1.0 – 1.0] | 1.0 [1.0 – 1.0] | 1.0 [1.0 – 1.2] | 0.62 |
| IL-10 (fold Δ [Q1, Q3]) | 18.3 [3.4 – 40.1] | 6.6 [1.6 – 34.2] | 22.9 [5.9 – 40.5] | 0.17 |
| IL-13 (fold Δ [Q1, Q3]) | 0.7 [0.3 – 1.5] | 0.8 [0.3 – 1.5] | 0.7 [0.3 – 1.6] | 0.72 |

**Supplemental Table S3. Comparison of MIS-C patients in the IVIG monotherapy and IVIG+anakinra groups (continued).** Statistical significance between IVIG monotherapy and IVIG+anakinra groups was determined by two-tailed Mann-Whitney test for continuous variables or by Fisher’s exact test where appropriate, and p-values <0.05 were considered significant. The IVIG+anakinra group includes patients who were treated both with CS and without CS. CV shock= cardiovascular, KD-like= Kawasaki disease-like, IQR= interquartile range, LDH= lactate dehydrogenase, INR= international normalized ratio, ALT= alanine aminotransferase, AST= aspartate aminotransferase, Cr= creatinine.

| **Parameter** | **+ CV shock** | **- CV shock** | **p-value** |
| --- | --- | --- | --- |
| Patients (n, %) | 20, 63% | 12, 38% | N/A |
| **MIS-C clinical features** |  |  |  |
| Non-specific (n, %) | 0, 0% | 6, 50% | N/A |
| CV shock (n, %) | 20, 100% | 0, 0% | N/A |
| KDSS-like (n, %) | 11, 55% | 0, 0% | N/A |
| **Laboratory findings on admission** [Q1, Q3] |  |  |  |
| Albumin, g/dL | 2.9 [2.3 – 3.4] | 3.1 [2.9 – 3.7] | 0.11 |
| ALT, units/L | 41 [17 – 75] | 38 [26 – 73] | 0.56 |
| AST, units/L | 44 [23 – 60] | 42 [31 – 61] | 0.63 |
| BNP, pg/mL | 3339 [1048 – 10462] | 1472 [133 – 3498] | 0.04 |
| Cr, mg/dL | 0.50 [0.39 – 0.88] | 0.45 [0.41 – 0.57] | 0.56 |
| CRP, mg/dL | 15.6 [12.0 – 20.1] | 12.1 [8.1 – 17.5] | 0.21 |
| D-dimer, mg/mL | 3.5 [2.1 – 4.1] | 3.3 [1.6 – 7.6] | 0.98 |
| Ferritin, ng/mL | 350 [287 – 589] | 273 [159 – 514] | 0.12 |
| Fibrinogen, mg/dL | 559 [433 – 672] | 516 [378 – 546] | 0.16 |
| INR, units | 1.2 [1.1 – 1.3] | 1.3 [1.2 – 1.4] | 0.02 |
| LDH, units/L | 286 [267 – 322] | 330 [225 – 366] | 0.63 |
| Lymphocytes, K/mcL | 0.89 [0.47 – 1.23] | 0.95 [0.59 – 1.53] | 0.48 |
| Neutrophils, K/mcL | 8.4 [5.5 – 13.5] | 5.3 [3.9 – 6.6] | 0.07 |
| Troponin, ng/mL | 0.05 [0.02 – 0.12] | 0.02 [0.02 – 0.02] | 0.03 |

**Supplemental Table S4. Comparison of MIS-C patients in the IVIG+anakinra group presenting with or without CV shock (continued).** Statistical significance between - CV shock and + CV shock groups was determined by two-tailed Mann-Whitney test for continuous variables or by Fisher’s exact test where appropriate, and p-values <0.05 were considered significant and shown in bold. The IVIG+anakinra group includes patients who were treated both with and without steroid. CV shock= cardiovascular, KD-like= Kawasaki disease-like, IQR= interquartile range, LDH= lactate dehydrogenase, INR= international normalized ratio, ALT= alanine aminotransferase, AST= aspartate aminotransferase, Cr= creatinine.


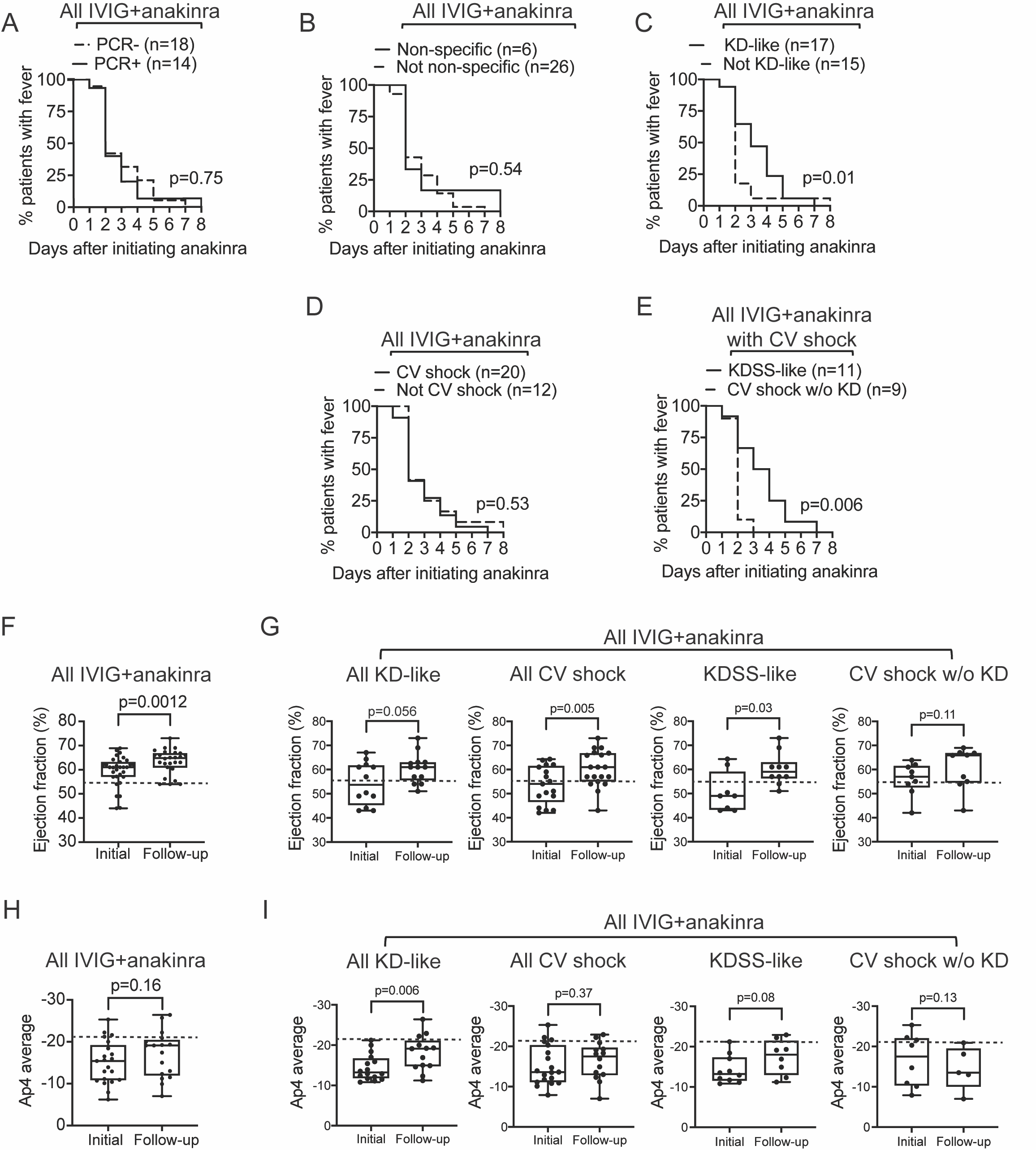


**Supplemental Figure** **S2. Clinical of MIS-C patients in the IVIG+anakinra group stratified by COVID PCR status and clinical phenotype.** Resolution of fever in all MIS-C patients in the IVIG+anakinra group discriminated by SARS-CoV-2 PCR status (A) or non-specific features (B), KD-like features (C), CV shock (D), and KDSS-like features (E) are shown. LVEF (F) and cardiac strain (H) at admission (initial) and follow-up of all MIS-C patients IVIG+anakinra group are shown, and subgroup analyses by clinical phenotypes are shown in G and I, respectively. The IVIG+anakinra group includes patients who were treated both with CS and without CS. The dotted lines denote the cut-off range for normal LVEF (55%) (F and G) and cardiac strain (Ap4 of -21.4) (H and I). Data were analyzed by log rank test (A and E) or non-parametric two-tailed Wilcoxon matched pairs signed rank test (F and I), and a p-value <0.05 was considered statistically significant.


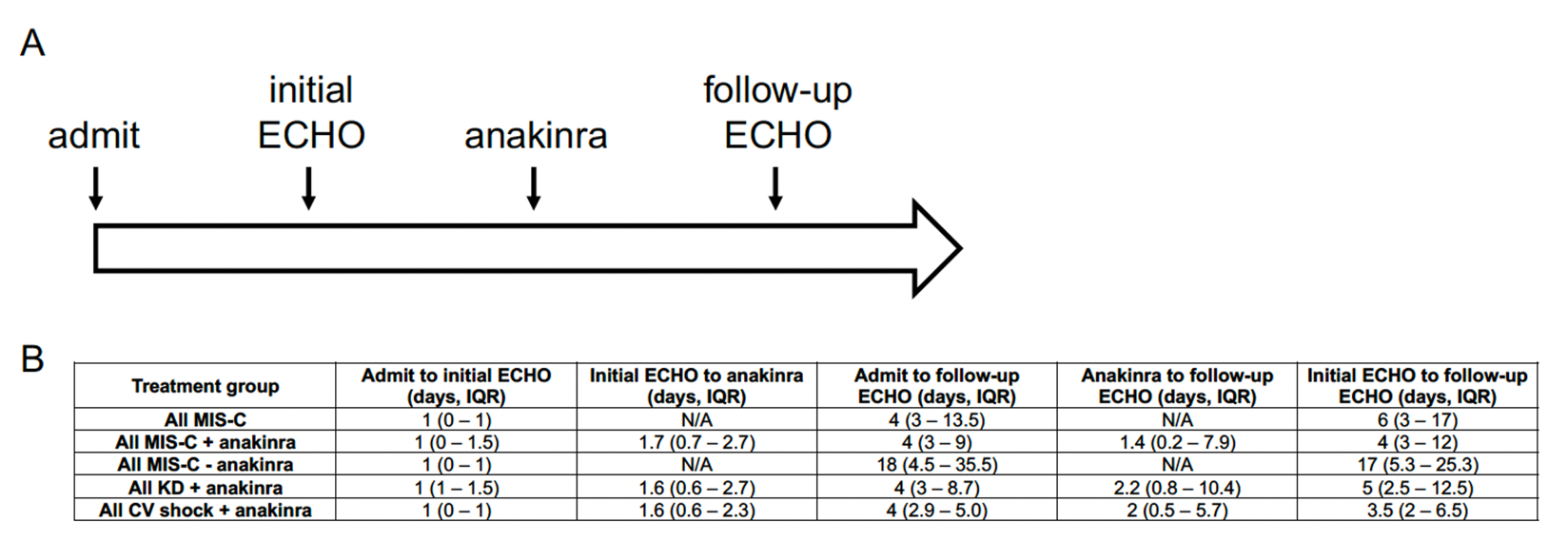


**Supplemental Figure S3. Timing of initial and follow-up echocardiograms in MIS-C patients.** A scheme of the various time measurements between admission and the follow-up echocardiograms (ECHO) are depicted in A. In B, the time intervals in days (expressed as median with Q1 – Q3) of the ECHOs relative to admission and initiation of anakinra in the various clinical phenotypes of MIS-C are shown. IQR= interquartile range.

| **Adverse event (n, %)** | **IVIG monotherapy**  **(n = 14)** | **IVIG+anakinra without CS**  **(n = 25)** | **IVIG+anakinra with CS (n = 9)** | **p-value** |
| --- | --- | --- | --- | --- |
| Neutropenia | 1, 7% | 5, 20% | 3, 33% | 0.36 |
| Grade 1 | 0, 0% | 2, 8% | 0, 0% | 0.41 |
| Grade 2 | 1, 7% | 1, 4% | 2, 22% | 0.25 |
| Grade 3 | 0, 0% | 2, 8% | 1, 11% | 0.54 |
| Grade 4 | 0, 0% | 0, 0% | 0, 0% | N/A |
| Rash | 0, 0% | 2, 8% | 0, 0% | 0.42 |
| Elevated LFTs | 2, 14% | 6, 24% | 7, 78% | 0.008 |
| < Grade 2 | 1, 7% | 2, 8% | 5, 55% | 0.02 |
| Grade 2 | 0, 0% | 2, 8% | 0, 0% | 0.41 |
| Grade 3 | 1, 7% | 2, 8% | 0, 22% | 0.47 |
| Grade 4 | 0, 0% | 0, 0% | 0, 0% | N/A |

**Supplemental Table S5.** Adverse events of severity of neutropenia and elevated LFTs stratified by severity according to CTCAE. The IVIG+anakinra group includes patients who were treated both with CS and without CS.
