# Supplementary material for: Clinical outcomes and safety of anakinra in the treatment of multisystem inflammatory syndrome in children: a single center observational study
Source: Pediatr Rheumatol Online J. 2023 Jul 31;21:76. doi: 10.1186/s12969-023-00858-z (PMC10388456; doi:10.1186/s12969-023-00858-z)
Supplement: Supplementary file 2 — Supplementary Material 2 [file 12969_2023_858_MOESM2_ESM.docx]

MIS-C Taskforce at Children’s National Hospital, Washington DC:

Infectious Diseases

Dr Roberta DeBiasi, Chief

Critical Care Medicine

Dr Michael Bell, Chief

Dr Matthew Sharron

Cardiology

Dr Charles Berul, Chief

Dr Ashraf Harahsheh

Dr Anita Krishnan

Rheumatology

Dr Sangeeta Sule, Chief

Dr Hemalatha Srinivasalu

Dr Tova Ronis

Dr Abbie Bosk

Hospitalist Medicine

Dr Karen Smith, Chief

Dr Kavita Parikh

Dr Jessica Herstek

Emergency Medicine

Dr Jay Pershad, Chief

Dr Jaclyn Kline

Hematology

Dr Suvankar Majumdar, Chief

Dr Yaser Diab

Immunology

Dr Hemant Sharma, Chief

Dr Vanessa Bundy

Pharmacy

Dr Eleanor Sadler

Gastroenterology

Dr Sona Sehgal

Neurology

Dr Beth Wells

Nephrology

Dr Marva Moxey-Mims, Chief

Dr Sun-Young Ahn

Pathology

Dr Meghan Delaney, Chief

Chief Medical Officer

Dr David Wessel
